# Virtual reality application matches the most established treatment for Mal de Debarquement Syndrome: A non-inferiority, randomized, open clinical trial

**DOI:** 10.1016/j.neurot.2024.e00390

**Published:** 2024-06-27

**Authors:** Catho Schoenmaekers, Dario De Smet, Choi Deblieck, Jan Van Riel, Andrzej Zarowski, Floris L. Wuyts

**Affiliations:** a Lab for Equilibrium Investigations and Aerospace, University of Antwerp, Antwerp, Belgium; b Telmio BV, Luchthavenlaan 27, Vilvoorde, Belgium; c European Institute for ORL-HNS, Sint-Augustinus Hospital, Wilrijk, Belgium

**Keywords:** Mal de Debarquement Syndrome, Perception of self-motion, Neuro-otological disorder, Virtual reality application, VOR re-adaptation treatment

## Abstract

Mal de Debarquement Syndrome (MdDS) is a debilitating neuro-otological disorder where individuals consistently feel self-motion, often triggered by motion like being on a boat (MT-MdDS). Due to the unknown pathophysiological mechanism, available treatment options for managing symptoms are limited. Our objective was to develop a virtual reality application (VRA) to simulate the full field optokinetic stimulation (OKS) booth and evaluate its efficacy compared to the standard treatment. In our randomized, open, non-inferiority clinical trial with 30 ​MT-MdDS patients, 15 received the OKS booth and 15 the new VRA over four consecutive days. Two 4-min treatment blocks were scheduled in the morning and afternoon, with a total of four blocks. Treatment effectiveness was evaluated through questionnaires and posturography. Our findings suggest that the choice of modality does not significantly differ in achieving an overall improvement in symptoms. We advocate that the VRA can be used as an accessible alternative to the booth method worldwide, effectively mitigating MdDS symptoms and enhancing the QoL of numerous MdDS patients.

## Introduction

Mal de Debarquement (MdD), commonly known as sea legs or land sickness, is a poorly understood neuro-otological condition, characterized by an oscillating perception of self-motion, especially noticeable when an individual is stationary. This perception of apparent self-motion can manifest as *side-to-side* swaying, *forward-backward* rocking, and/or *up-down* bobbing, which may persist continuously or for a significant portion of the day. Patients may also experience a gravitational pull, which is the feeling of being pushed or pulled to a specific side. Many patients experience a sensation akin to walking on ‘a trampoline or the moon’ during locomotion. When symptoms persist for longer than a month after the onset, it is denoted as Mal de Debarquement Syndrome (MdDS) [1]. Typically, MdDS is triggered after being exposed to passive motion, e.g., after a journey on a boat, but can also be triggered in the absence of such a passive motion event. This observation led to a preliminary classification of MdDS into motion-triggered (MT-MdDS) and spontaneous or other onset MdDS (non-MT MdDS) [2]. Regarding non-MT MdDS, in accordance with the Barany Society's terminology guidelines, it should be described as non-motion triggered, motion-moderated, non-spinning vertigo. However, for brevity and convenience, we will refer to this condition as non-MT MdDS [2]. MdDS stands apart from other vestibular disorders due to its unique characteristic: symptoms temporarily alleviate when individuals are re-exposed to passive motion, e.g., during a car ride [2]. The disorder may be aggravated by various factors, such as exposure to busy environments, visual stimuli, or fatigue, making daily life exceedingly challenging for those affected. Several patients stop working due to the condition, since MdDS itself drains a lot of energy.

Whereas vertigo commonly arises from a measurable peripheral dysfunction of the vestibular organ, conventional vestibular tests fail to detect any irregularities in MdDS patients. As a result, it is hypothesized that MdDS has its roots in the central nervous system (CNS), specifically linked to an inadequate adjustment to passive motion [3]. Neuroimaging studies report altered functional connectivity, metabolic activity, and gray matter volume in patients with MdDS compared to healthy controls [3,4]. The prevailing hypothesis suggests that this syndrome stems from a maladaptation of the velocity storage mechanism, a component influencing the vestibulo-ocular reflex (VOR) pathways [5]. Over the course of 30 years, extensive research has delved into the spatial characteristics of velocity storage [[6], [7], [8], [9], [10], [11], [12], [13]], with a crucial discovery highlighting the activation of the velocity storage mechanism through optokinetic stimulation (OKS) [10].

Building upon this finding, Dai and colleagues developed an OKS paradigm that is now regarded as the most established in the treatment of MdDS, with the argument that a treatment protocol based upon the re-adaptation of the VOR relieves the perception of self-motion [5,14]. They treated a total of 24 patients with MdDS who were asked to watch a rotating full-field visual stimulus inside an OKS booth while the investigators rolled the patient's head side-to-side [5]. This treatment engages the visual pathways, which transmit their input to the velocity storage in the vestibular nuclei, and subsequently to the inferior olivary nucleus and cerebellum [10]. Of the 24 patients, 16 showed remission or partial recovery, i.e., response >50%, six were initially better, and one did not respond to the treatment. Hence, Dai and colleagues showed that the readaptation of the VOR led to a mean 70% improvement in the perception of self-motion. Another study conducted by Dai and colleagues, involving 141 patients (122 females and 19 males), demonstrated that after a year of OKS treatment, significant improvement was maintained in 52% of MT-MdDS patients and 48% of non-MT MdDS patients, with full remission observed in 27% of MT-MdDS and 19% of non-MT MdDS patients. This shows that OKS treatment produces a long-lasting effect and significantly mitigates MdDS symptoms [14]. In 2017, our group performed a sham controlled study to replicate the method of Dai and colleagues in 25 patients (13 ​MT-MdDS and 12 non-MT MdDS) [22]. Building further on this approach, we administered 131 OKS treatments in 51 ​MT- and 50 non-MT MdDS patients [15]. All treatments were carried out in the full field-of-view OKS booth, following a more stringent protocol that involved maintaining a fixed head roll at 0.167 ​Hz on three consecutive days. The overall success rate was 64.1%, with no significant difference between MT-MdDS (64.2%) and non-MT MdDS (63.3%). These studies confirm the evidence that VOR readaptation treatment provides relief for two-thirds of patients suffering from MdDS, irrespective of the onset type.

However, one of the main limitations of OKS treatment is that the OKS booth is immobile, bulky, and restricted in access. A previous study by Yakushin and colleagues investigated the efficacy of limited-visual-field goggles as a substitute for full-field optokinetic stimulation in MdDS [16]. They treated five female patients, comprising four MT-MdDS and one non-MT MdDS, using a stripes program for OKS implemented through the Google Daydream Viewer. All five patients showed immediate improvement post-treatment, persisting at the 2-month follow-up. Symptoms returned in one patient. Based on these findings, we developed a virtual reality application (VRA) tailored for Android smartphones. It features complete field optokinetic stripes that consistently move horizontally, irrespective of the device's orientation and tilt. This simulates the prototypical stripes experienced in a OKS booth during head rolls. The current study presents a non-inferiority, randomized (1:1), open-label clinical trial designed to asses whether our “remote” VRA using our fixed paradigm offers therapeutic benefits similar to those provided by the OKS booth [15]. Thirty patients with MT-MdDS were randomly assigned to the VRA condition or the OKS booth condition. The primary outcome measures included the visual analogue scale (VAS) and static posturography measures.

## Material and Methods

### Informed consent

Patients provided their written informed consent before participation in this clinical trial, approved by the ethical committees of the Gasthuis Zusters Antwerpen (GZA, 210407ACADEM) hospitals and the Antwerp University hospital (UZA, B3002021000057) in accordance with the Declaration of Helsinki and its amendments. Prior to enrolment, patients gave their written informed consent. The term ‘participants’ will be used further to refer to the patients who were enrolled in the clinical trial.

### Inclusion and exclusion criteria

Inclusion criteria comprised: 1) Participants must have a confirmed diagnosis of MdDS; 2) Participants have not started any new medication (including oral contraception) in the past month; 3) Participants have never used the virtual reality application before; 4) Participants are a male or female between the ages of 18–80, and have a WHO performance status between 0 and 1; 5) Female participants are not pregnant or do not wish to become pregnant during the study enrolment; 6) Female participants must not breastfeed during the clinical trial.

### Participants

The study enrolled its first and last MT-MdDS patients on September 21, 2021, and December 1, 2023, respectively. A total of 30 ​MT-MdDS patients were included and were randomly assigned to receive treatment with either the VRA or the OKS booth. Our study population comprised 21 females (mean age: 48 ​± ​9), and nine males (mean age: 38 ​± ​12). The VRA group consisted of nine females (mean age: 52 ​± ​12) and six males (mean age: 41 ​± ​11). The OKS booth group comprised 12 females (mean age: 45 ​± ​10) and three males (mean age: 32 ​± ​14). The average symptom duration (SD) for the VRA group is 3 ​± ​4 years, and for the booth group 2 ​± ​2 years.

Before receiving treatment, MdDS patients were diagnosed by a MdDS specialist at the European Institute for Otorhinolaryngology at GZA Sint-Augustinus Hospital in Wilrijk, Belgium. The assessments involved the use of the SO STONED anamnesis, encompassing Symptoms, Often, Since, Trigger, Otology, Neurology, Evolution, and Duration. This comprehensive evaluation across these dimensions contributed to establishing a distinctive profile for this specific vestibular disorder [17]. In this study, the “Since” and “Trigger” questions were specifically tailored to address MdDS characteristics based on the diagnostic criteria published by the Barany Society [2]. For example, participants were asked whether their MdDS symptoms began after passive transportation, e.g., cruise, and whether they experienced symptom improvement when re-exposed to passive transportation, as these are key indicators of MdDS.

### Randomized non-inferiority clinical trial set-up

Participants were randomly assigned to either the VRA or the OKS booth group. The allocation was determined by their anonymized study number, i.e., participants with an even number were placed in the VRA group while participants with an odd number were assigned to the OKS booth group. Before receiving treatment, all participants underwent an initial hearing and equilibrium assessment, including oculomotor, rotation, and water caloric tests in order to rule out any potential vestibular impairments.

The treatment for MdDS remained uniform regarding parameters and session numbers, irrespective of VRA or OKS booth treatment. Both treatments were based on the OKS protocol proposed by Dai and colleagues, involving a combination of head-roll movements while the participant was exposed to a moving stripe pattern at a velocity of 10° per second, known as the OKS stripes [5,15]. As previously noted, the optokinetic stripes in the VRA maintained a vertical alignment relative to Earth regardless of the phone's orientation when tilting the head. The stripes adjusted in counter-tilt to match head movements, creating the illusion that they remained vertically oriented and unaffected by the head's tilt. The stripes in both the VRA and the booth were black and white. However, the stripes of the OKS booth are not as sharp because they are projected, making them less defined than those on the VRA. The stripes of the VRA are defined using WebGL, a high-speed, high-resolution low-level web library, with RGBA colors: vec4(1.0,1.0,1.0,1.0) for white and vec4(0.0,0.0,0.0,1.0) for black. The last number indicates intensity, set to the maximum. The brightness of the phone could be set to the preference of the participant. As a result, the actual intensity was determined by the phone's brightness. The physical width of the stripes depends on the phone and is expressed in pixels, using 10% of the screen height multiplied by the device pixel ratio for the highest resolution possible. There are thirteen black stripes on a white background visible in both the booth and VRA, ensuring a similar immersive experience. In the booth, participants were positioned at the center and instructed to keep their gaze fixed forward, akin to a steady stare, allowing the stripes to move past without actively tracking them, simulating the experience of viewing from a moving train. As a result, participants experienced complete immersion in the OKS booth, similar to the VRA, as they did not perceive any discernible reference frame at the top or bottom of the booth during the session. With the VRA, when patients look up or down, the entire field of view was filled with stripes, devoid of any reference points.

A qualified investigator simultaneously oscillated the participant's head manually from the left to the right shoulder within a range of ±20°. Due to the fact that the resonance frequency of the vestibular system is 0.167 ​Hz, the head was oscillated from left to right at precisely that frequency [18]. An auditory cue, presented as a tone ladder with an ascending and descending melody of a 6-s period, served as a guide reference.

All participants were submitted to the OKS treatment on four consecutive days. On each day, two OKS blocks, lasting for 4 ​min, were scheduled in the morning and afternoon, with a total of four blocks per day. The inter-block-interval comprised approximately 5 ​min and the inter-session-interval approximately 3 ​h. Before each treatment session, the direction of the OKS stripes was determined using the Fukuda stepping test and/or the participant's subjective perception of their internal oscillation. During the Fukuda stepping test, participants were instructed to close their eyes and march in place with their arms extended forward for 45 ​s. The Fukuda test was performed twice daily, once before each morning and afternoon treatment session. If the participant's direction of rotation during the stepping test was >15°–20°, the test was considered positive, and the OKS stripes moved in the opposite direction. For instance, if the participant exhibited a deviation to the left, the stripes would move from left to right. If the Fukuda stepping test was negative, meaning no clear deviation (or a deviation of <15°) was perceived, the direction of the stripes was determined based on the participant's description of their perceived motion or the direction of their gravitational pull. If neither deviation during the Fukuda test nor a description of movement was present, we used a leftward direction. This decision was based on a previous study by our team, which showed that patients who responded best to the treatment were treated with stripes moving from right to left [15]. The option of using either vertical or horizontal stripes was deliberately avoided due to the technical constraints of our OKS booth, which only allows for movement from left to right or right to left. The stripes in the booth are projected on an optokinetic drum that rotates with a motor mounted on the ceiling of the booth. The orientation of the axis of the motor was fixed and unable to tilt 90° to generate vertical motion of the stripes. In order to maintain consistency between both modalities, we exclusively used the left and right motion of the stripes.

The follow-up phase extended for up to four weeks after the OKS treatment to assess the post-treatment effects on both the VRA and OKS booth groups.

### Outcome measures

**Fig. 1:**
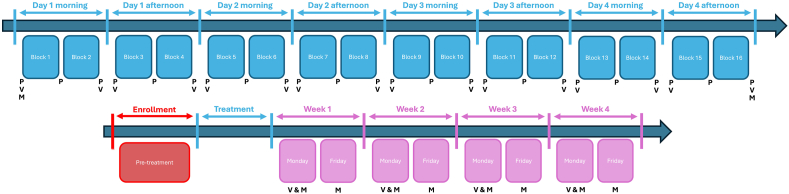
Schematic overview of clinical trial set-up and the various measurements conducted. In blue, the four consecutive treatment days, in red the enrolment or pre-treatment phase, and in pink the four weeks of the follow-up phase. The measurements include P for posturography, V for VAS score, and M for MdDS symptom questionnaire.

Both subjective and objective measurements were selected to assess the treatments' efficacy (Fig. 1). Subjective outcome measures comprised the following: 1) primary outcome measure, the visual analogue scale (VAS) score to assess symptom severity and the impact of treatment, 2) secondary outcome measure, a questionnaire focussing on MdDS symptoms. The MdDS questionnaire addressed symptoms of fatigue, headache, eye strain, difficulties in focussing, increased salivation, sweating, nausea, brain fog (lack of focus and mental clarity), blurry vision, perception of self-motion with eyes open, perception of self-motion with eyes closed, vertigo, problems with orientation, and uncomfortable feeling in the stomach. Participants had to indicate the severity of these symptoms on the first day (prior to the first session) and last day (after the final session) of the treatment, as well as every Monday and Friday throughout the four weeks of the follow-up phase, totalling 10 assessments. The VAS score indicated the participant's subjective experience of self-motion, the hallmark symptom of MdDS. Participants were asked to rate the intensity of their perception of self-motion by putting a vertical dash on a 10 ​cm horizontal line, with 0 indicating no disturbance and 10 maximal perception of self-motion. The VAS score was evaluated both before and after each treatment block, alongside assessments every Monday throughout the four-week follow-up phase, totalling 20 assessments.

For the objective outcome measure, we employed static posturography as a second primary outcome measure, utilizing a Wii Balance Board (Wii BALANCE BOARD. RVL-021. Nintendo Co., Ltd. 11-1 Kamitoba-Hokotate-cho Minami-ku. Kyoto, 601-8501 Japan) to assess postural control and stability. This functional evaluation is crucial for understanding the interaction between different sensory systems, including the visual, vestibular, and somatosensory systems. The balance board measures the participant's frequency and velocity of movement by means of four pressure sensors, located under the support points of the board. In normal participants, postural sway is also present, with values ranging from 3.17 ​mm for lateral sway amplitude to 1.44 ​mm for sagittal/lateral sway ratio [20]. In MdDS, heightened sway and sway ratios are evident due to their oscillatory sensations of self-motion. This increased sway prompts patients to shift their center of pressure more towards one side of the board. Consequently, the pressure sensors on that side of the board will detect a rise in weight. At a sampling rate of 100 ​Hz, the Wii Balance Board demonstrates a sensitivity of 69.39% and specificity of 73.16% in detecting balance dysfunction [21]. Participants were instructed to stand on the Wii-balance board with their arms crossed in front of their chests and with eyes closed for 1 ​min.

### Statistical analysis

The posturography data were analyzed using a software program derived from the Colorado University Wii Balance Board code, which was developed at the Neuromechanics Laboratory within Colorado University (https://www.colorado.edu/neuromechanics/research/wii-balance-board-project). Following data acquisition, a Butterworth filter of fourth order with a cut-off frequency of 0.17 ​Hz was applied to the data using MATLAB (Release 2023a, developed by The MathWorks, Inc., Natick, Massachusetts, United States) [22]. Utilizing a customized MatLab routine, we were able to compute the confidence ellipse area (CEA) with a 95% confidence interval (CI), representing the region containing 95% of the posturography measurements. The MatLab routine employed a discrete Fourier transformation (DFT) to generate a power spectrum illustrating the frequency of movement in both the medio-lateral (ML) and antero-posterior (AP) directions. The DFT discerns the frequency content of a time-domain signal. Subsequently, the area under the curve (AuC) of these power spectra, serving as a measure of energy content, was calculated for both ML and AP directions, denoted as AuC-ML and AuC-AP.

Regarding The VAS scores, they were used to determine subjective improvement from pre- to post-treatment and calculate the response rate as follows:

(i) Response rate pre- to post-treatment ​= ​$\left(\frac{\text{preVAS}-\text{postVAS}}{\text{preVAS}}\right)*100\%$.
(ii) Response rate pre-treatment to follow-up ​= ​$\left(\frac{\text{preVAS}-\text{folVAS}}{\text{preVAS}}\right)*100\%$.

When a VAS score improvement of 50%–75% was observed, we classified it as a partial response. VAS score improvement of 76%–100% was classified as full response or remission. Improvement ranging from 0% to 49% was categorized as non-responsive. Any decrease lower than 0% was classified as worsening after treatment. Paired sample *t*-test was used to investigate if there was a difference in response rate between modality, pre- to post-treatment and pre-treatment to follow-up.

All statistical analyses were conducted in JMP® (version Pro 16, SAS Institute Inc, Cary, NC, 1989–2001), with a significance threshold set at alpha ​= ​0.05 at a confidence level of 95%. To evaluate the overall impact of the OKS treatment from pre- to post-treatment, and to follow-up, we used four linear mixed models (LMMs), each dedicated to one of the outcome measures or dependent variables (VAS scores, CEA, AuC-ML, and AuC-AP). Employing a stepwise backward approach, we initially constructed a comprehensive model that encompassed all relevant independent variables, including fixed effects and interaction terms. Subsequently, through a series of steps, we systematically removed non-significant interaction terms and fixed effects, resulting in an optimized model that effectively captures the significant factors explaining the data. The dependent variables in these models were the VAS scores, CEA, AuC-ML, and AuC-AP. The fixed effects incorporated in all models as independent variables were symptom duration, timepoint (pre- and post-treatment), gender (male - female), age, and modality (VRA or OKS booth). To address non-independence among observations from the same participant, we introduced the participant number as a random intercept in all models. The validity of the models was ensured by examining residuals for normality and homoscedasticity. Descriptive statistics were used to gain insights into the dataset and study population, including parameters such as age and gender. Additionally, non-parametric Wilcoxon tests were used to identify significant differences in specific symptoms (e.g., brain fog) from pre- to post-treatment, and to follow-up. A Mann-Whitney *U* test was applied to assess whether modality (VRA or OKS booth) had an impact on the pre- to post-treatment, and to follow-up, difference in symptom severity. A paired sample *t*-test was used to investigate significant differences in response rate between modalities, pre- to post-treatment and pre-treatment to follow-up. To address multiple comparisons, a Benjamini-Hochberg correction was applied for the Wilcoxon and Mann-Whitney U tests using RStudio (version 1.2.1335). All reported results are provided with standard errors.

## Results

### The virtual reality application yields comparable benefits compared to the most established OKS treatment

**Fig. 2:**
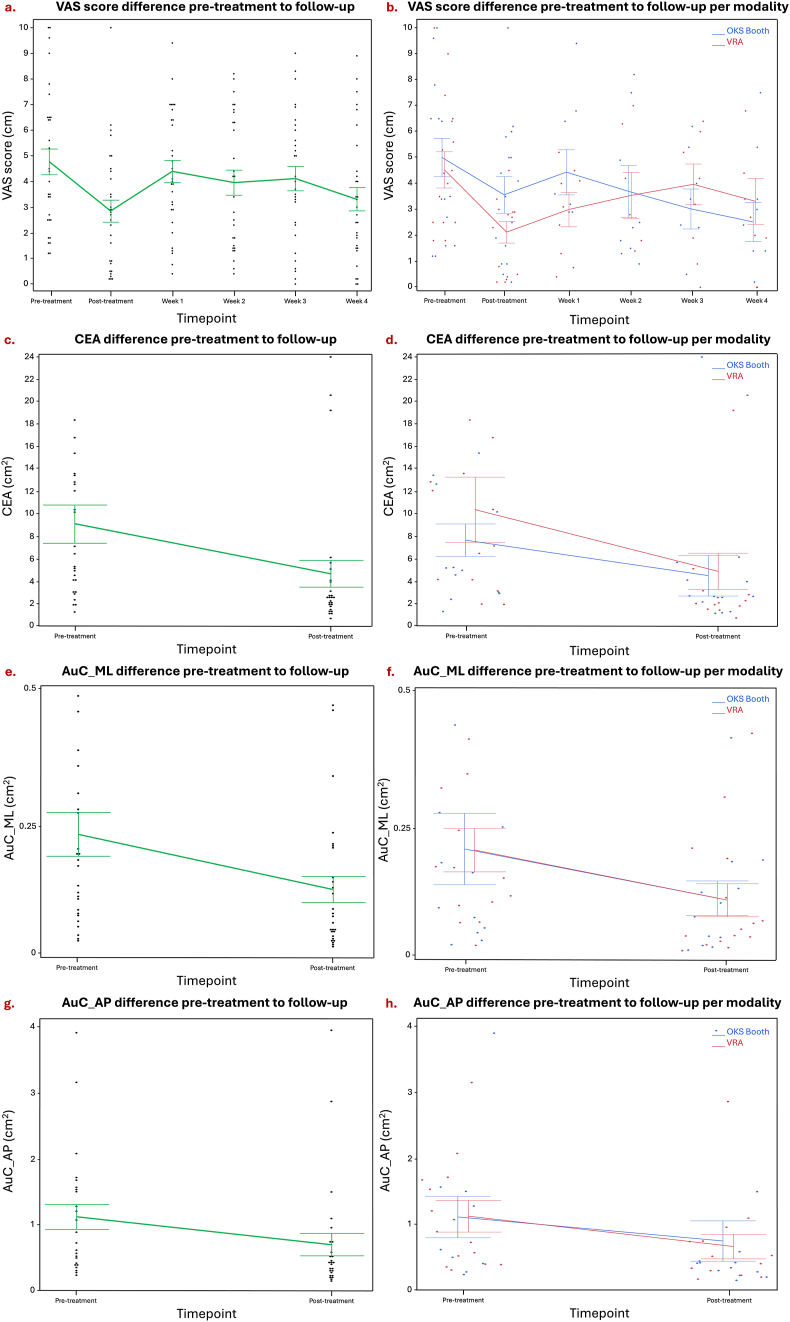
Illustrating pre-treatment to follow-up improvement: on the visual analogue scale (VAS) on average **(a)**, and per modality **(b)**, on the confidence ellipse area (CEA) on average **(c)**, and per modality **(d)**, on the area under the curve for the medio-lateral movement on average **(e)**, and per modality **(f)**, and on the area under the curve for the anterior-posterior movement on average **(g)**, and per modality **(h)**. In blue, OKS booth represents the most established treatment, and in red, the VRA denotes the virtual reality application. Error bars ±1 SD. Week x represents the number of weeks of the follow-up phase.

The primary model, with VAS as dependent variable with an intercept of 2.85 ​± ​0.46 ​cm (p ​< ​0.0001), showed a significant effect of timepoint (pre- to post-treatment) (p ​< ​0.0001), indicating that the pre-treatment VAS score was 1.92 ​± ​0.42 ​cm higher compared to the post-treatment VAS score. There was no significant effect observed for the used modality, indicating no significant difference in VAS score improvement pre- to post-treatment between the VRA and the booth. Additionally, we investigated the VAS difference between pre-treatment and follow-up, intercept 3.31 ​± ​0.48 ​cm (p ​< ​0.0001), as well as post-treatment and follow-up. There remained a significant difference (p ​= ​0.0005) between pre-treatment and four weeks follow-up, with a 1.46 ​± ​0.37 ​cm higher pre-treatment score, indicating sustained treatment effect four weeks later without variance between modalities. No significant difference (p ​= ​0.8073) was found between post-treatment and follow-up, again suggesting consistent improvement maintained over the four-week follow-up period (Fig. 2).

The secondary modal, with CEA as dependent variable with an intercept of 4.54 ​± ​1.42 ​cm^2^ (p ​= ​0.0027), showed a significant effect of timepoint (pre- to post-treatment) (p ​= ​0.0034), indicating that the pre-treatment CEA was 4.52 ​± ​1.40 ​cm^2^ higher compared to the post-treatment CEA. The third model, with AuC-ML as dependent variable with an intercept 0.10 ​± ​0.03 ​cm^2^ (p ​= ​0.0010), showed a significant effect of timepoint (pre- to post-treatment) (p ​= ​0.0168), indicating that the pre-treatment AuC-ML was 0.10 ​± ​0.04 ​cm^2^ higher compared to the post-treatment AuC-ML. The fourth model, with AuC-AP as dependent variable with an intercept of 0.93 ​± ​0.48 ​cm^2^ (p ​= ​0.590), showed a significant effect of timepoint (pre- to post-treatment) (p ​= ​0.0178), indicating that the pre-treatment AuC-AP was 0.94 ​± ​0.37 ​cm^2^ higher compared to the post-treatment AuC-AP. There was no significant effect observed for the used modality, indicating no significant difference in posturography improvement pre- to post-treatment between the VRA and the booth. All estimates can be found in Table 1.

**Table 1:** Overview of the estimates and *p*-values of the different linear mixed models (LMM). Significance threshold set at alpha = 0.05.

Fixed effects	Estimate (cm)	Std error (cm)	CI 95% (cm)	*P*-Value
***Model 1a: the effect of optokinetic stimulation on the subjective improvement pre- to post-treatment, visual analogue scale (VAS score), in MdDS patients***				
Intercept Timepoint (pre)	2.85 1.92	0.46 0.42	[1.91; 3.78] [1.06; 2.79]	**<0.0001** **<0.0001**
***Model 1b: The effect of optokinetic stimulation on the subjective improvement pre-treatment to follow-up, visual analogue scale (VAS score), in MdDS patients***				
Intercept Timepoint (pre)	3.31 1.46	0.48 0.37	[2.34; 4.28] [0.70; 2.21]	**<0.0001** **0.0005**
***Model 2: The effect of optokinetic stimulation on the objective improvement, confidence ellipse area (CEA), in MdDS patients***				
Intercept Timepoint (pre)	4.54 4.52	1.42 1.40	[1.67; 7.41] [1.64; 7.41]	**0.0027** **0.0034**
***Model 3: The effect of optokinetic stimulation on the subjective improvement, area under the curve for medio-lateral movement (AuC-ML), in MdDS patients***				
Intercept Timepoint (pre)	0.10 0.10	0.03 0.04	[0.04; 0.17] [0.02; 0.17]	**0.0010** **0.0168**
***Model 4: The effect of optokinetic stimulation on the subjective improvement, area under the curve for anterior-posterior movement (AuC-AP), in MdDS patients***				
Intercept Timepoint (pre)	0.93 0.94	0.48 0.37	[-0.04; 1.89] [0.18; 1.70]	0.0590 **0.0178**
*Timepoint (post-treatment) was taken as the reference value for model 1a, 2, 3, and 4* *Timepoint (follow-up) was taken as the reference value for model 1b*				

CI, confidence interval.

### Effect of the virtual reality application on the spectrum of MdDS symptoms

Since MdDS patients show a broad spectrum of symptoms, we used a symptom specific questionnaire to investigate the impact of the OKS treatment. We used three Wilcoxon tests to investigate the symptom improvement i) pre- to post-treatment, ii) pre-treatment to follow-up, and iii) post-treatment to follow-up (Table 2). The first Wilcoxon, pre- to post-treatment, after applying Benjamini-Hochberg correction, showed significant improvement for all symptoms, except for tired eyes, increased salivation, sweating, and stomach discomfort. The second Wilcoxon, pre-treatment to follow-up, after applying Benjamini-Hochberg correction, showed significant improvement, except for increased salivation and vertigo. The third Wilcoxon, post-treatment to follow-up, after applying Benjamini-Hochberg correction, showed no significant differences. Additionally, by using a Mann-Whitney *U* test (Table 2), the onset type did not influence the improvement of symptoms except for blurry vision (U ​= ​281.00, z ​= ​2.05, p ​= ​0.0407) and perception of self-motion with eyes closed (U ​= ​284.00, z ​= ​2.15, p ​= ​0.0318). Notably, the VRA group exhibited greater improvement in blurry vision post-treatment, with an average difference of 2.40 ​± ​2.44 ​cm compared to the booth group's average difference of only 0.33 ​± ​2.50 ​cm. Similarly, concerning the perception of motion with eyes closed, the VRA group demonstrated greater improvement post-treatment, with an average difference of 2.80 ​± ​2.11 ​cm compared to the booth group's average difference of 1.07 ​± ​2.09 ​cm. However, after applying the Benjamini-Hochberg correction, the differences in blurry vision and the perception of self-motion with eyes closed were no longer statistically significant between modalities. Therefore, it is advisable to interpret these results with caution.

**Table 2:** Results from the Wilcoxon Signed Rank tests evaluating alterations in subjective improvement.

Domain	Test Statistics S	P-Value	Adjusted P-Value	Mann-Whitney U	Z	P-Value	Adjusted P-Value
***1) Pre- to post-treatment***							
Fatigue	−105.50	**0.0264**	**0.0386**	226.50	−0.23	0.8179	0.9246
Headache	−104.00	**0.0276**	**0.0386**	212.50	−0.82	0.4123	0.9246
Tired eyes	−69.50	0.1480	0.1727	243.00	0.42	0.6725	0.9246
Troubles focussing	−121.00	**0.0097**	**0.0226**	246.50	0.56	0.5730	0.9246
Increased salivation	−39.50	0.2773	0.2773	238.50	0.31	0.7583	0.9246
Sweating	−86.00	**0.0472**	0.0601	234.50	0.07	0.9450	0.9450
Nauseous	−141.50	**0.0006**	**0.0028**	240.50	0.34	0.7351	0.9246
Brain fog	−135.50	**0.0033**	**0.0116**	249.50	0.69	0.4911	0.9246
Blurred vision	−122.50	**0.0071**	**0.0199**	281.00	2.05	**0.0407**	0.2849
Perception of self-motion with eyes open	−187.00	**<0.0001**	**0.0007**	275.50	1.78	0.0752	0.3509
Perception of self-motion with eyes closed	−183.00	**<0.0001**	**0.0007**	284.00	2.15	**0.0318**	0.2849
Vertigo	−107.50	**0.0146**	**0.0261**	228.00	−0.18	0.8586	0.9246
Orientation problems	−110.00	**0.0149**	**0.0261**	247.00	0.61	0.5435	0.9246
Stomach discomfort	−61.50	0.1711	0.1843	226.50	−0.25	0.8039	0.9246
***2) Pre-treatment to follow-up***							
Fatigue	−160.00	**<0.0001**	**0.0003**	243.00	0.42	0.6730	1.0000
Headache	−97.50	**0.0392**	**0.0457**	245.50	0.53	0.5985	1.0000
Tired eyes	−163.50	**0.0002**	**0.0005**	235.00	0.08	0.9325	1.0000
Troubles focussing	−156.00	**0.0004**	**0.0008**	213.50	−0.78	0.4372	1.0000
Increased salivation	−12.00	0.7436	0.7436	214.00	−1.01	0.3136	1.0000
Sweating	−101.50	**0.0276**	**0.0351**	220.00	−0.52	0.6052	1.0000
Nauseous	−115.00	**0.0063**	**0.0098**	232.50	0.00	1.0000	1.0000
Brain fog	−175.00	**<0.0001**	**0.0003**	223.50	−0.36	0.7187	1.0000
Blurred vision	−169.50	**<0.0001**	**0.0003**	260.50	1.18	0.2393	1.0000
Perception of self-motion with eyes open	−202.50	**<0.0001**	**0.0003**	263.00	1.27	0.2030	1.0000
Perception of self-motion with eyes closed	−186.00	**<0.0001**	**0.0003**	242.00	0.38	0.7051	1.0000
Vertigo	−60.50	0.1858	0.2001	230.50	−0.07	0.9469	1.0000
Orientation problems	−106.00	**0.0195**	**0.0273**	261.00	1.22	0.2237	1.0000
Stomach discomfort	−111.50	**0.0054**	**0.0095**	238.50	0.26	0.7911	1.0000
***3) Post-treatment to follow-up***							
Fatigue	−61.50	0.1862	0.8507	253.50	0.85	0.3927	0.6665
Headache	−14.00	0.7759	0.8507	251.50	0.78	0.4372	0.6665
Tired eyes	−123.00	**0.0079**	0.1106	231.00	−0.04	0.9665	0.9700
Troubles focussing	−32.00	0.5165	0.8507	205.50	−1.11	0.2681	0.6665
Increased salivation	29.00	0.3991	0.8507	218.00	−0.83	0.4059	0.6665
Sweating	−12.50	0.7703	0.8507	239.00	0.29	0.7732	0.9021
Nauseous	4.50	0.9138	0.9138	218.50	−0.67	0.5042	0.6665
Brain fog	−28.00	0.5698	0.8507	6.40	−0.84	0.4034	0.6665
Blurred vision	16.50	0.7254	0.8507	192.00	−1.75	0.0795	0.5565
Perception of self-motion with eyes open	43.50	0.3757	0.8507	212.00	−0.84	0.4027	0.6665
Perception of self-motion with eyes closed	16.00	0.7422	0.8507	188.00	−1.87	0.0615	0.5565
Vertigo	43.00	0.3622	0.8507	234.00	0.04	0.97	0.9700
Orientation problems	12.50	0.7899	0.8507	247.50	0.64	0.5237	0.6665
Stomach discomfort	−49.00	0.2328	0.8507	259.00	1.29	0.1979	0.6665

Subjective experiences were measured across 14 symptoms using a VAS score. Additionally, Mann–Whitney U tests were conducted to assess whether the changes in subjective improvement differed between both modalities. Adjusted P-values were calculated using the Benjamini–Hochberg correction for multiple comparisons.

### Response rate of the virtual reality application in the treatment of Mal de Debarquement Syndrome

In terms of full response before and after treatment, 20% of all participants showed a full response averaging 88% (0.88 ​± ​0.06) improvement. Specifically, within the VRA group, 33% of participants showed a full response, with an average of 87% (0.87 ​± ​0.06) improvement, while in the booth group, 7% of participants showed a full response, averaging 94% (0.94 ​± ​0.03) improvement. When considering a full response from pre-treatment to follow-up, 20% of all participants showed an average improvement of 90% (0.90 ​± ​0.09). Specifically, within the VRA group, 20% of participants showed a full response, on average 89% (0.89 ​± ​0.10) improvement, while in the booth group, 20% of participants showed a full response, with an average of 91% (0.91 ​± ​0.11) improvement.

In terms of partial response before and after treatment, 27% of all participants showed a partial response, on average 65% (0.65 ​± ​0.08) improvement. Specifically, within the VRA group, 27% of participants exhibited a partial response, with an of average 66% (0.66 ​± ​0.07) improvement, while in the booth group, 27% of participants showed a partial response, averaging 63% (0.63 ​± ​0.10) improvement. When considering the partial response rate from pre-treatment to follow-up, 3% of all participants showed a partial response, averaging 63% (0.63 ​± ​0.01) improvement. Specifically, within the VRA group, no participant showed a partial response, while in the booth group, 7% of the participants showed a partial response, with an average of 63% (0.63 ​± ​0.01) improvement.

Further, 53% of all participants were non-responsive to either treatment, on average 28% (0.28 ​± ​0.14) improvement. Specifically, 40% of participants of the VRA group and 67% of the booth group were non-responders with an average improvement of 24% (0.24 ​± ​0.11) and 31% (0.31 ​± ​0.16), respectively. When considering non-responsiveness from pre-treatment to follow-up, 77% of all participants did not respond, showing a mean improvement of 22% (0.22 ​± ​0.14). Specifically, within the VRA group, 80% of participants were non-responders, with an average of 23% (0.23 ​± ​0.16) improvement while 73% of participants in the booth group showed to be non-responders, averaging 22% (0.22 ​± ​0.12) improvement.

No significant difference was found between modality regarding response rate, pre- to post-treatment (p ​= ​0.2639), and pre-treatment to follow-up (p ​= ​0.9267).

## Discussion

In this non-inferiority, randomized (1:1), open clinical design trial, we assessed if limited-field OKS generated by the VRA has the potential to provide clinical efficacy equivalent to the most established treatment, i.e., the full-field-of-view OKS booth, in treating MdDS. Our findings demonstrate the therapeutic efficacy of VRA and the OKS booth, lasting for at least up to four weeks, without a significant difference observed between modalities.

The activation of the velocity storage system is thought to be most effectively achieved through full-field-of-view OKS, primarily due to its efficiency in inducing a rotational sensation of self-motion or vection [5,16,23]. Previous studies argued that vection is crucial in treating MdDS, directly related to the activation of the velocity storage mechanism, and can only be induced through activation of peripheral vision [5,10,24,25]. Therefore, it was speculated that restricted visual field-of-view (i.e., VRA) may not be as effective as a full field-of-view OKS (i.e., OKS booth) in treating MdDS. However, in the current study, peripheral vision remained inactive. Despite this, the treatment administered by the VRA proved to be equally effective, showing no significant difference from the OKS booth. This suggests that the velocity storage mechanism was activated despite a restricted field-of-view, indicating that vection and peripheral vision activation might not hold crucial significance in treating MdDS as argued by Yakushin and colleagues [16].

Our data indicate that the type of modality does not differ in improving VAS, CEA, and AuC (ML/AP) scores or measures from pre- to post-treatment, pre-treatment to follow-up, and post-treatment to follow-up. However, it is worth noting that the VRA exhibited a significant difference compared to the booth in terms of improving blurry vision post-treatment, showing a mean difference of 2.40 ​± ​2.44 ​cm versus the booth group's average difference of only 0.33 ​± ​2.50 ​cm. Similarly, regarding self-motion perception with eyes closed, the VRA group showed improvement post-treatment, with a mean difference of 2.80 ​± ​2.11 ​cm compared to the booth group's mean difference of 1.07 ​± ​2.09 ​cm. However, after the Benjamini-Hochberg correction for multiple comparisons was applied, the previously significant findings lost their significance. Consequently, it is advised to interpret these results cautiously. It should be emphasized that the VRA was designed to closely resemble the OKS booth, though certain differences remain. Notably, the stripes in the OKS booth are less defined and the brightness could not be adjusted to the participant's preference compared to those in the VRA. It has been shown before that the brightness has an effect on how well MdDS patients tolerate the stimuli. This distinction is important, as the enhanced visibility of stimuli in the VRA may lead to a greater impact, given that the stimuli are more prominently displayed and thus potentially more effective. Conversely, Murata and colleagues observed that reduced brightness to 2 lux was better tolerated by MdDS patients compared to 3 lux [26]. This is important to take into account for future studies and treatment protocols.

The direction of the stripes can be easily adjusted horizontally from left to right or right to left, as well as vertically from bottom to top or top to bottom. In contrast to the VRA, the booth's motion capabilities are limited. Consequently, our study was restricted to using only horizontally moving stripes. Nonetheless, the similar response rate of the VRA suggests the effectiveness of horizontally moving stripes, providing the first evidence of their therapeutic value. Similarly, it is plausible that showing vertical motion through the VRA could equally enhance symptom improvement by facilitating more personalized treatments tailored to the individual patients. Based on the response rate categorization by Dai's calculations to facilitate comparison between studies [14], 60% of participants that received the VRA showed a response pre- to post-treatment, with 33% showing full remission (75–100%). Participants that received the booth, 34% showed a response pre- to post-treatment, with 7% showing full remission. It should be noted that in the clinical practice, a response rate of 30% is already considered clinically significant. Following this categorization, 73% participants from the VRA group showed a response pre- to post-treatment, with 33% showing full remission. Similarly, 78% participants in the booth group showed a response pre- to post-treatment, with 7% showing full remission. Surprisingly, despite reports suggesting that scrolling on a computer screen or simply looking down on a cell phone screen often triggers MdDS symptoms, participants in the VRA group experienced no discomfort while using the application [14]. To the contrary, a significant number reported an improvement in visual sensitivity.

Patients suffering from MdDS are offered to receive VOR treatment using the OKS booth at GZA Sint-Augustinus, Antwerp, Belgium. Over a span of two years and encompassing 131 three-day-treatments, our retrospective study revealed that only 39% of all patients originated from Belgium [15]. Other MdDS patients participated from various other countries, including the Netherlands, United States of America, Canada, Brazil, Norway, Sweden, Denmark, Germany, United Kingdom, France, Hungary, Austria, Poland, Portugal, and Spain. Patients are often driven to receive the recognition and validation they seek, along with access to potential treatment, despite the risks associated with travelling, which is known as one of the major triggers of MdDS symptoms. Our study demonstrated that the VRA could potentially offer a long-awaited solution to alleviate persistent symptoms in MdDS. Patients can access the VRA remotely, receiving online guidance and support in using the VRA, effectively reducing the risk of travel-related symptom retriggering upon their return home. It remains to be elucidated whether home-based VRA therapy is as efficacious as controlled VRA treatment, considering that patients will be responsible for performing the head rolls themselves in sync with the rhythm of the melody.

In conclusion, MdDS is a poorly understood neuro-otological condition characterized by an oscillating perception of self-motion accompanied by a broad spectrum of secondary symptoms (e.g., brain fog). While the precise cause of onset remains unclear, previous theories suggest it may originate from maladaptive changes in the velocity storage mechanism and recommend the use of full field-of-view OKS as an optimal way to activate velocity storage effectively. Unfortunately, the conventional full field-of-view OKS booth, while effective, poses limitations due to its bulkiness, cost, and limited accessibility. Our study shows that the VRA is equally effective, as evidenced by the lack of significant difference in response rate between the VRA and the OKS booth. We argue that the VRA holds promise as a globally accessible alternative to the OKS booth, effectively alleviating MdDS symptoms and enhancing the QoL for numerous MdDS patients. Future studies using larger sample sizes are needed to enhance generalizability.

## Author contribution

C.S., F.L.W. and A.Z. contributed to the conception of this study; C.S. contributed to data acquisition; C.S., D.D.S and C.D. contributed to data analysis; C.S., D.D.S., C.D. and F.L.W. contributed to data interpretation; J.V.R. contributed to development of virtual reality application; C.S. contributed to drafting the paper; and C.S., D.D.S., C.D., and F.L.W. contributed to a substantial revision of the manuscript.

## Declaration of competing interest

The authors declare that they have no known competing financial interests or personal relationships that could have appeared to influence the work reported in this article.

## Funding

This study was funded by the Belgian Science Policy (Prodex), ESA-AO-2004-093. Catho Schoenmaekers is a research assistant of the 10.13039/501100003130Research Foundation Flanders (Belgium, FWO-Vlaanderen, Grant 1SF9122 ​N and 1SF9124 ​N).

## Ethical approval

This study was approved by the ethical committees of the Gasthuis Zusters Antwerpen (GZA, 210407ACADEM) hospitals and the Antwerp University hospital (UZA, B3002021000057) in accordance with the Declaration of Helsinki and its amendments.

## Acknowledgments

The authors would like to thank all medical professionals of the Ear-Nose-Throat (ENT) department of the Sint-Augustinus hospital for diagnosing and referring the MdDS patients. And, all MdDS patients who were willing to participate in the clinical trial.

## Availability of data and material

Data can be requested from C.S. (pending scientific review and a completed material transfer agreement). Requests for the data should be submitted to catho.schoenmaekers@uantwerpen.be. Requests for usage of virtual reality application, please contact floris.wuyts@uantwerpen.be.

## Consent to participate

A written informed consent was obtained for all participants before their inclusion in the study.

## Consent for publication

All authors have approved the version to be published.
